# 环状RNA在肺癌中的研究进展

**DOI:** 10.3779/j.issn.1009-3419.2020.102.38

**Published:** 2020-12-20

**Authors:** 祥伟 葛, 智博 张, 晓玉 智, 进良 汪

**Affiliations:** 100853 北京，解放军总医院第一医学中心肿瘤内科 Department of Oncology, the First Medical Center of Chinese PLA General Hospital, Beijing 100853, China

**Keywords:** 环状RNA, 肺肿瘤, 诊断, 治疗, 预后, Circular RNA, Lung neoplasms, Diagnosis, Treatment, Prognosis

## Abstract

肺癌是全球发病率和死亡率最高的恶性肿瘤，其较差的预后结局为患者带来了沉重的负担。目前肺癌诊疗面临的形势依然严峻，亟待探寻有效的治疗靶点及分子标志物。环状RNA（circular RNA, circRNA）是共价闭合的非编码RNA，因其保守性、稳定性、组织特异性等生物学特性而备受关注。诸多研究发现环状RNA通过吸附miRNA等多种机制参与肺癌的调控，并对肺癌的早期诊断、治疗及预后评价发挥重要作用。近年来，circRNA在肺癌的相关研究层出不穷。本文就目前circRNA在肺癌诊断、治疗以及预后方面的进展予以归纳总结。

## Abstract (en)

Lung cancer is the most common malignant tumor with the highest morbidity and mortality worldwide, and its imposes an insupportable burden on patients due to its poor prognosis. The diagnosis and treatment of lung cancer is under great pressure. Therefore, it is urgent to explore effective therapeutic targets and molecular markers. Circular RNA (circRNA) is a kind of covalently closed non-coding RNAs, which has attracted much attention due to its conservation, stability and tissue specificity. Many studies have found that circRNA participates in the regulation of lung cancer through various mechanisms such as sponging miRNA and plays a part vital role in the early diagnosis, treatment and prognosis evaluation. In recent years, there have been numerous studies on circRNA in lung cancer. This paper summarizes the current progress of circRNA in the diagnosis, treatment and prognosis of lung cancer.

肺癌是目前世界范围内最常见的恶性肿瘤疾病，对人类生命健康造成严重威胁。据统计，2018年全球约有210万肺癌新增病例和180万肺癌死亡病例，发病率和死亡率居所有癌种中首位^[1]^。根据组织学分型，肺癌可以分为小细胞肺癌（small cell lung cancer, SCLC）和非小细胞肺癌（non-small cell lung cancer, NSCLC）两种，其中SCLC和NSCLC分别约占肺癌总数的15%和85%^[2]^。尽管临床诊断和治疗手段均有所改善，但由于诊断不及时、受益人群有限、患者耐药等原因，肺癌的5年生存率仍不乐观。另外，缺乏相对特异性的肿瘤标志物为肺癌的诊治及预后增添了挑战。因此，有必要深入研究肺癌分子机制以探寻肺癌潜在的生物标志物及治疗靶点。

环状RNA（circular RNA, circRNA）是一种特殊的内源性非编码RNA。早在20世纪70年代circRNA就被发现存在于RNA病毒中^[3]^。但介于当时技术的局限，circRNA被认为是剪接过程的副产物，因而并未受到广泛关注^[4]^。近些年随着高通量测序技术和生物信息学的发展，circRNA被大量发现并逐渐成为RNA领域的研究热点。目前，诸多研究证实circRNA能够参与肺癌发生发展过程的调控，并有望为肺癌的诊断、治疗及预后提供新思路^[5, 6]^。

## 1 circRNA的生物学特性及功能

circRNA是共价闭合的非编码RNA分子，在真核生物转录组中广泛存在。通常根据circRNA的来源将其分为：外显子circRNAs（ecRNAs）、内含子circRNA（ciRNAs）以及外显子-内含子circRNA（EIciRNAs）^[7]^。其中以外显子circRNAs最为常见。与线性RNA不同，circRNA没有5’端的帽子结构和3’端的多聚腺苷酸尾，能够抵抗核酸外切酶RNase R的降解，因而circRNA相较于线性RNA稳定性好且半衰期更长^[8]^。研究^[9]^还发现，circRNA表现出良好的物种保守性。另外，circRNA的表达具有组织特异性和发育阶段特异性，提示circRNA可能参与机体多种病理生理过程的调控^[10]^。

circRNA的功能研究多集中在以下几个方面：①作为分子海绵吸附miRNA。竞争性内源RNA（competing endogenous RNA, ceRNA）机制指出具有相同miRNA应答元件（miRNA response elements, MREs）的RNA能够竞争性结合miRNAs，从而相互调控影响彼此的表达^[11]^。目前，多数circRNA研究围绕miRNA分子海绵这一机制展开; ②通过结合RNA聚合酶II调控亲本基因的转录^[12]^; ③与RNA结合蛋白相互结合发挥生物学作用^[9]^; ④翻译蛋白。例如Yang等^[13]^发现Circ-FBXW7编码蛋白进而抑制胶质瘤发生。

## 2 circRNA与肺癌的诊断

早期且准确的诊断对肺癌的治疗至关重要。尽管已有多种诊断方法应用于临床，但由于费用、准确度以及患者接受程度等原因，当前的手段仍有改进空间。因此对肺癌诊断标志物的探索依然很有必要。circRNA具有保守性、稳定性、特异性等优势，因而具有成为肺癌新兴标志物的潜能^[14]^。一项针对中国肺癌人群的*meta*分析汇集了8项关于肺癌组织和血液中circRNA诊断效能的研究显示，纳入的circRNA总灵敏度和特异度分别为0.77和0.76，总受试者工作特征曲线（receiver operating characteristic curve, ROC）的曲线下面积（area under curve, AUC）为0.78，提示circRNA在中国肺癌人群中具有诊断潜能^[15]^。

### 2.1 肺癌组织circRNA的诊断价值

Wang等^[16]^发现，在区分NSCLC和正常组织方面，hsa_circ_0077837和hsa_circ_0001821的AUC分别为0.921和0.863，展现了这两种circRNA对肺癌的诊断价值。Liu等^[17]^证实hsa_circ_11780在NSCLC组织和细胞系中表达均显著下降，且低表达hsa_circ_11780的患者出现较大肿瘤（> 3 cm）、远处转移和较差生存预后的风险更大。Zhao等^[18]^对61对配对的肺癌和癌旁组织进行分析，发现hsa_circ_0037515和hsa_circ_0037516在NSCLC中低表达，二者的AUC分别为0.81和0.82，同样表现出较好的诊断能力。而联合hsa_circ_0037515和hsa_circ_0037516后的总AUC提升至0.90，提示肺癌组织circRNA联合诊断的重要性。

### 2.2 血液circRNA的诊断价值

相比于传统活检，液体活检具有操作简单、侵入性小、成本低等优点，因此研究前景广阔。目前已有文献初步证实血浆circRNA具有较好的诊断能力，如circRNA-002178^[19]^、circMAN1A2^[20]^等。Chen等^[21]^通过高通量测序技术以识别肺腺癌（lung adenocarcinoma, LUAD）患者血浆外泌体中差异表达的circRNA。与对照组相比，105个circRNA表达升高，78个circRNA表达降低。进一步研究发现，hsa_circ_0001492和hsa_circ_0001346在LUAD早期即表达明显上调，而对照组血浆几乎检测不到，提示hsa_circ_0001492和hsa_circ_0001346可能成为早期LUAD诊断候选标记物。

Liu等^[22]^检测分析hsa_circ_0005962和hsa_circ_0086414在LUAD患者血浆中差异表达。二者联合诊断AUC达到0.81，提示双circRNA可能作为诊断LUAD的非侵入性生物标志物。另外，血液circRNA可能与肿瘤进展有关，LUAD患者术后hsa_circ_0005962表达较术前明显下降。而hsa_circ_0086414的表达水平与表皮生长因子受体（epidermal growth factor receptor, *EGFR*）突变相关。与野生型患者相比，*EFGR*突变型患者hsa_circ_0086414高表达。该研究表现了血液circRNA多方面的应用价值。当然，为实现血液circRNA肺癌诊断的临床转化，尚需更大的样本量以及更为深入的机制探索。

## 3 circRNA与肺癌的治疗

既往研究发现circRNA能够作为调控分子促进或者抑制肺癌的发生发展，因而调控circRNA的表达水平对肺癌的恶性生物学行为具有重要意义。目前，多项研究依据circRNA的ceRNA机制进行肺癌恶性生物学行为的机制探索（表 1）。例如，Yao等^[23]^发现circGFRA1在肺癌细胞内表达升高，并通过circGFRA1/miR-188-3p/PI3K/AKT通路促进肺癌的恶性增殖。LIMK1作为一种丝苏氨酸蛋白激酶，通过影响肌动蛋白细胞骨架参与上皮间质转化（epithelial-mesenchymal transition, EMT），调控肿瘤进程^[24]^。Qin等^[25]^发现circ_0012673在肺癌组织和细胞系中高表达。circ_0012673海绵吸附miR-320a导致下游靶蛋白LIMK1表达升高，从而抑制肺癌细胞凋亡，促进其增殖、迁移和EMT进程。

**表 1:** circRNA通过ceRNA机制作用于肺癌恶性生物学行为的总结 Summary of circRNA acting on malignant biological behaviors of lung cancer through ceRNA mechanis

circRNA	Dysregulation	Cell lines	Function	Sponge target	Ref.
circGFRA1	Up	A549, H838	Proliferation (+)	miR-188-3p	[23]
circ-0000211	Up	A549, H1299, H1650	Migration (+), invasion (+)	miR-622	[26]
circ-ABCB10	Up	A549, H292	Proliferation (+), migration (+)	miR-556-3p	[27]
circ_0000326	Up	A549, H1299	Proliferation (+), apoptosis (-), migration (+)	miR-338-3p	[28]
circ_0014130	Up	PC-9, A549	Proliferation (+), apoptosis (-), invasion (+)	miR-136-5p	[29]
circ-SOX4	Up	A549, SPC-A1	Proliferation (+), migration (+), invasion (+)	miR-1270	[30]
circCCDC66	Up	A549, H1299	Proliferation (+), apoptosis (-), migration (+), invasion (+)	miR-33a-5p	[31]
circ_0012673	Up	A549, H23	Proliferation (+), apoptosis (-), migration (+), EMT (+)	miR-320a	[25]
circCDR1as	Up	A549, Calu-3	Proliferation(+), apoptosis (-), migration (+), invasion (+)	miR-219a-5p	[32]
circ_0058124	Up	A549, H1975	Proliferation (+), apoptosis (-), migration (+), invasion (+)	miR-1297	[33]
circ-MTO1	Down	A549, SPC-A1	Proliferation (-)	miR-17	[34]
cMras	Down	A549, H1299	Proliferation (-), migration (-)	miR-567	[35]
circ-IGF1R	Down	PC9, A549	Migration (-), invasion (-)	miR-1270	[36]
circCRIM1	Down	A549, H1299, SPC-A1	Migration (-), invasion (-)	miR-93, miR-182	[37]
circ_0007059	Down	A549, H1975	Proliferation (-), EMT (-)	miR-378	[38]
circ_11780	Down	A549, H226	Proliferation (-), migration (-), invasion (-)	miR-544a	[17]
circ_0006427	Down	SPC-A1, Calu-3	Proliferation (-), migration (-), invasion (-)	miR-6783-3p	[39]
circPTPRA	Down	H23, H1755, H522	Migration (-), invasion (-), EMT (-)	miR-96-5p	[40]
circSMARCA5	Down	A549	Proliferation (-), migration (-), invasion (-)	miR-19b-3p	[41]
circ_0002483	Down	A549, H1299	Proliferation (-), migration (-), invasion (-)	miR-182-5p	[42]
circ_0078767	Down	A549, H23	Proliferation (-), apoptosis (+), invasion (-)	miR-330-3p	[43]
circRNA: circular RNA; EMT: epithelial-mesenchymal transition; ceRNA: competing endogenous RNA.					

### 3.1 circRNA与肺癌耐药

随着抗肿瘤药物的不断问世，为肺癌患者带来了更多希望，但是耐药问题依然是困扰临床治疗的一大难题。因此，亟待对肺癌耐药机制进行深入探索以探寻高效的生物标志物或治疗靶点。研究发现，部分circRNA能够参与肺癌的耐药进程（表 2）。Hong等^[44]^研究发现，circCPA4作为let-7的分子海绵，其下调可以影响程序性死亡配体1（programmed death-ligand 1, PD-L1）使其表达降低，进而抑制NSCLC细胞的生长、迁移和EMT过程。另外，NSCLC来源的含PD-L1的外泌体能够促进其干细胞特性，增强NSCLC细胞对顺铂的耐受性。Li等^[42]^报道circ_0002483能够降低miR-182-5p表达水平，解除其对靶分子GRB2、FOXO1、FOXO3的抑制，进而增强NSCLC对紫杉醇的敏感性。circRNA_103762在肺癌中高表达，通过抑制靶蛋白CHOP诱导肺癌的多药耐药^[45]^。

**表 2:** 肺癌circRNA对抗肿瘤药物敏感性影响的总结 Summary of the effects of circRNA on tumor drug sensitivity

CircRNA	Cell lines	Drugs	Sensitivity	Ref.
circAKT3	A549, H1299	Cisplatin	Down-regulated	[5]
circ-ABCB10	A549, H292	Cisplatin	Down-regulated	[27]
circZFR	A549, H522	Cisplatin	Down-regulated	[46]
circ_0076305	A549, H1650	Cisplatin	Down-regulated	[47]
circ_0004015	A549, HCC827	Gefitinib	Down-regulated	[48]
circ_0003998	A549, H1299	Docetaxel	Down-regulated	[49]
circ_0002483	A549, H1299	Paclitaxel	Up-regulated	[42]
circ_0001946	A549	Cisplatin	Up-regulated	[50]
circESRP1	H69, H446	Cisplatin, etoposide	Up-regulated	[51]
circ-SMARCA5	H1299, H1437	Cisplatin, gemcitabine	Up-regulated	[52]

### 3.2 circRNA与肺癌免疫治疗

肿瘤细胞能够表达多种机制来逃避免疫系统的攻击，为自身的生长创造条件。程序性死亡受体1（programmed death protein 1, PD-1）是一种跨膜蛋白，已被发现几乎表达于各种类型的肿瘤细胞表面，并通过于PD-L1相互作用参与肿瘤的免疫逃逸机制^[53]^。近些年，以PD-1/PD-L1为靶点的免疫检查点抑制剂（immune checkpoint inhibitors, ICIs）为肺癌治疗提供了强大武器。Wang等^[19]^研究发现，circRNA-002178在肺腺癌组织中异常高表达，通过吸附miR-34促进肺癌细胞PD-L1的表达。同时，肺癌细胞能够分泌外泌体circRNA-002178并传递给T细胞，通过抑制miR-28-5p进而促进T细胞PD-1的表达。文献^[54]^证实，CXCR4参与细胞毒性T淋巴细胞耗竭、诱导抗PD-1药物耐药等过程。Zhang等^[55]^发现circFGFR1/miR-381-3p/CXCR4通路通过促进肺癌细胞对抗PD-1治疗药物的耐药进程，进而发挥免疫抑制效应。提示circRNA能够参与肿瘤免疫逃逸机制，相关通路抑制剂的联合使用有望提高临床疗效，为肿瘤的免疫疗法提供新的思路。

## 4 circRNA与肺癌的预后

肺癌患者的预后监测是评价临床诊疗效果的关键环节，对调整用药方案、改善患者生存时间有重要意义。研究证实，多种circRNA能够作为肺癌患者独立预后指标，与肺癌患者生存期紧密相关，如circSMARCA5^[52]^、circ_11780^[17]^、circCRIM1^[37]^。Liu等^[17]^对93例NSCLC患者肿瘤组织进行RT-qPCR检测发现hsa_circ_11780异常低表达，而低表达hsa_circ_11780的患者往往肿瘤更大，伴有远处转移以及更严重的肿瘤原发灶-淋巴结-转移（tumor-node-metastasis, TNM）分期。以*Kaplan-Meier*法进行生存分析表明低表达hsa_circ_11780的NSCLC患者总生存期（overall survival, OS）更短。circHIPK3来源于染色体11p13区的癌基因*HIPK3*的2号外显子。Chen等^[6]^发现敲低circHIPK3能够抑制NSCLC细胞株A549、H838和H1299增殖、迁移、侵袭能力，并诱导自噬的发生，而circHIPK3和linHIPK3对自噬的调控作用相互拮抗，circHIPK3:linHIPK3（C:L）比值能够反映肿瘤细胞的自噬水平。对于晚期NSCLC患者来说，高C:L比值（> 0.49）又是其低生存率的有效指标。这些结果提示自噬调节因子circHIPK3具有作为预后因子的潜在临床应用价值。

EGFR酪氨酸激酶抑制剂（EGFR-tyrosine kinase inhibitor, EGFR-TKIs）是*EGFR*敏感突变的NSCLC患者的重要治疗选择。Liu等^[56]^通过对使用EGFR-TKI吉非替尼后有效组与无效组NSCLC患者血浆circRNA测序，检测到1, 377个差异表达的circRNA。RT-qPCR检测证实hsa_circ_0109320与hsa_circ_0134501在吉非替尼有效组中高表达。进一步研究发现hsa_circ_0109320的高表达与患者更好的无进展生存期（progression-free survival, PFS）相关，提示hsa_circ_0109320可能是反映吉非替尼疗效的生物标志物。Fu等^[57]^发现hsa_circRNA_012515在NSCLC的组织、细胞尤其是吉非替尼耐药的细胞株中表达显著升高。另外，hsa_circRNA_012515表达上调与患者淋巴结转移、肿瘤分期及预后密切相关。高表达hsa_circRNA_012515的NSCLC患者其OS和PFS更短。研究者还发现与I期/II期NSCLC患者相比，hsa_circRNA_012515在III期/IV期患者中的表达水平更高。由此可见，hsa_circRNA_012515具有良好的临床相关性，可能是预测NSCLC患者不良预后的生物标志物。

## 5 展望

随着研究的不断深入，circRNA与肺癌的联系正在日益凸显。一方面，circRNA作为促癌或抑癌因子调控肺癌的增殖、转移、凋亡、自噬等生物学行为，调节化疗或靶向药物的敏感性以及免疫治疗疗效，为辅助临床治疗提供初步理论基础; 另一方面，组织或血液中circRNA的差异表达在肺癌的早期诊断及预后评估都展现了一定的相关性，有望成为肺癌潜在的生物标志物。但是目前circRNA研究尚处于早期阶段，多数研究者将重点置于miRNA海绵吸附功能探索，许多机制尚未阐明。其临床相关性研究也局限于少量样本，其转化价值有待商榷。相信未来circRNA领域将会有更多突破，为肺癌诊疗提供更多思路。
